# The Role of Mitochondria in Reactive Oxygen Species Generation and Its Implications for Neurodegenerative Diseases

**DOI:** 10.3390/cells7120274

**Published:** 2018-12-17

**Authors:** Saima Kausar, Feng Wang, Hongjuan Cui

**Affiliations:** 1 State Key Laboratory of Silkworm Genome Biology, Southwest University, Beibei, Chongqing 400716, China; drkausarsn@hotmail.com (S.K.); fengwang_swu@163.com (F.W.); 2 Engineering Research Center for Cancer Biomedical and Translational Medicine, Southwest University, Beibei, Chongqing 400716, China; 3 Chongqing Engineering and Technology Research Center for Silk Biomaterials and Regenerative Medicine, Southwest University, Beibei, Chongqing 400716, China

**Keywords:** electron transport chain, mitochondria, neurodegenerative diseases, oxidative damage

## Abstract

Mitochondria are dynamic cellular organelles that consistently migrate, fuse, and divide to modulate their number, size, and shape. In addition, they produce ATP, reactive oxygen species, and also have a biological role in antioxidant activities and Ca^2+^ buffering. Mitochondria are thought to play a crucial biological role in most neurodegenerative disorders. Neurons, being high-energy-demanding cells, are closely related to the maintenance, dynamics, and functions of mitochondria. Thus, impairment of mitochondrial activities is associated with neurodegenerative diseases, pointing to the significance of mitochondrial functions in normal cell physiology. In recent years, considerable progress has been made in our knowledge of mitochondrial functions, which has raised interest in defining the involvement of mitochondrial dysfunction in neurodegenerative diseases. Here, we summarize the existing knowledge of the mitochondrial function in reactive oxygen species generation and its involvement in the development of neurodegenerative diseases.

## 1. Introduction

Mitochondria are important cellular organelles that control various vital physiological processes in the bodies of organisms. Being a major consumer of oxygen, mitochondria utilize approximately 98% of the total amount of inhaled oxygen. Mitochondria efficiently generate energy that is required for almost all types of cellular activities; this energy is also used to contract both voluntary and involuntary muscles [1,2]. Additionally, the produced energy is used to sustain ionic gradients across the plasma membrane, which are essential for the excitability of excitable cells, permitting the accumulation of secreted material into vesicles, and allowing for the vesicle fusion and cycling essential for the secretion of neurotransmitters [3]. Thus, a mitochondrial functional disorder causes different disorders, such as alterations in tissue functions that may manifest as disease, as well as major defects in tissue function that may lead to handicap or death. In fact, mitochondria are necessary in the bodies of animals not just to provide energy, but also to maintain other physiological activities of cells, such as cellular calcium signaling, and any defect in this process might lead to dysfunction and disease [4,5].

The respiratory chain in mitochondria is a greatly efficient system. It catalyzes alternating one-electron oxidation reduction reactions that predisposes each carrier of an electron to side reactions with oxygen. It has been shown that mitochondria are the major intracellular source of reactive oxygen species under normal physiological conditions [6,7]. According to estimates, 1–2% of total daily oxygen consumption goes to reactive oxygen species production, and a woman with an average weight of 60 kg generates 160–320 mmol of free radicals each day from cellular respiration, while a man of 80 kg produces almost 215–430 mmol per day [8].

Neurodegenerative diseases are a group of heterogeneous disorders with discrete clinical symptoms and genetic etiologies. The diseases are characterized by the progressive loss of physiologically or anatomically associated neuronal systems. Parkinson’s disease and Huntington’s disease are typical examples of neurodegenerative diseases [9]. In spite of this heterogeneity, mitochondrial dysfunction is considered to be a unifying basic mechanism involved in different types of neuronal degeneration. Mitochondrial dysfunction has widespread detrimental consequences for cellular functions; for instance, it leads to impaired energy generation, impaired cellular calcium buffering, the activation of phospholipases and proteases, nitric oxide synthase, and the production of reactive oxygen species [10]. Thus, they play a crucial role in ageing, and can directly interact with variety of specific proteins that are thought to be involved in genetic forms of neurodegenerative disorders [11,12]. Furthermore, there are several lines of evidence that suggest that mitochondrial dysfunction is linked with neurodegenerative disorders [10]. However, our understanding is limited regarding the different mechanisms by which mitochondrial dysfunction influences physiological functions. Here, our main focus is to review recent data on the role of mitochondria in reactive oxygen species and its deleterious impact on the progression of neurodegenerative diseases.

## 2. The Structure and Functions of Mitochondria

A mitochondrion is a double-membraned, semi-autonomous cellular organelle that is separated from the cytoplasm of a cell by the mitochondrial membranes. The outer mitochondrial membrane is spongy in nature, which allows for free cross-movement of small, uncharged molecules and ions through the porin [13,14]. However, larger molecules, particularly proteins, have to be transported inside the mitochondrion via special translocases [15]. The inner membrane is highly impermeant and forms the main barrier between the mitochondrial matrix and the cytosol. The space between the outer and inner mitochondrial membranes is called the intermembrane space and harbors a variety of different proteins, which seem to be involved in different physiological functions of the cell. The inner membrane has at least three morphologically discrete subregions, including cristae, cristae junctions, and boundary membranes. These structures alter their shape in response to the cell’s metabolic needs and stress [16,17].

The structure of cristae appears to differ greatly between different tissues, and the functional importance of these variations in the structure of cristae remains largely mysterious [4]. Different groups of researchers have attempted to generate detailed three-dimensional reconstructions of cristae and to model the bioenergetic consequences of altering the shape of their structure [18,19,20]. Some researchers have correlated the complex structure of cristae with their energy demands; for instance, Perkins et al. [18] compared the structure of cristae in the mitochondrion of rod and cone cells, and observed greater cristae connectivity, narrower crista junctions, and almost 3-fold more surface area for cristae membranes in cone cells compared to that in rods. Further, they suggested that, as cones and rods utilize a different bioenergetic signature, the aerobic ATP demand and production is higher in cones than in rods. Hence, cones use two different strategies to enhance their aerobic ATP production: increasing the number of mitochondria and increasing the surface area of cristae membranes. However, it must be noted that we still have little exact information regarding the functional importance of the complex features of, and structural variations in, mitochondria.

Mitochondria regulate various biological processes in a cell for its survival and precise functioning. They functionally control the production of energy, the electron transport chain, cell signaling, apoptosis, and programmed cell death, while their functional disorder has serious health consequences. Considering the involvement of mitochondria in the key processes of cells as well as their health impacts, researchers all over the world are attempting to explore the role of mitochondrial dysfunction in the pathogenesis of different diseases [3,21,22].

## 3. Mitochondrial Sites for the Generation of Free Radicals

**Figure 1:**
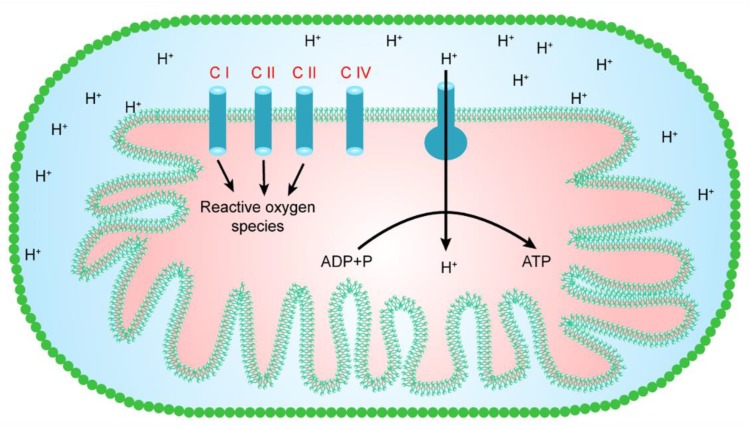
The major sites for the production of reactive oxygen species in a mitochondrion.

Reactive oxygen species are produced in various cellular compartments. However, mitochondria are a major contributor to reactive oxygen species, as they generate almost 90% of the total number of cellular reactive oxygen species [23]. Hence, mitochondria seem to represent one of the key sources of reactive oxygen species production in the majority of cell types. However, mitochondria in different cells/tissues may clearly differ in their capacity to generate free radicals using various substrates, and this capacity of the mitochondria may depend on the composition of a membrane, the age of an organism, and the species of animal. In a mitochondrion, at least eight sites are known to be involved in the production of reactive oxygen species. However, as shown in Figure 1, mitochondrial complexes I, II, and III mainly lie within the respiratory chain, and are thought to make a major contribution to the generation of reactive oxygen species [24,25]. Oxidative phosphorylation is the main process that produces unpaired electrons. These unpaired electrons interact with O_2_, resulting in the production of greatly reactive free radicals (superoxide ions). The superoxide ions are converted into other reactive oxygen species, such as H_2_O_2_ and hydroxyl ions (-OH) [4].

## 4. Complex I and the Generation of Free Radicals

Mitochondrial complex I, also known as NADH CoQ reductase, catalyzes the electron transfer from NADH to ubiquinone, which is accompanied by the movement of protons from the mitochondrial matrix to the intermembrane space [26,27]. During the 20th century, it was shown that mitochondrial complex I is involved in the generation of reactive oxygen species [24]. Additionally, it is believed that mitochondrial complex I is a key source of reactive oxygen species and is a major contributor to cellular oxidative stress. Hence, since then, an enormous literature has been developed on the mechanism by which reactive oxygen species are produced from complex I. For instance, the transfer of an electron at complex I may cause the production of reactive oxygen species, whereas rotenone, a complex I inhibitor, seems to suppress the generation of reactive oxygen species, presumably by preventing the electron’s passage further into the distal end where reactive oxygen species are produced [4,28]. Cadenas et al. [8] suggested that water-soluble co-enzyme Q homologs are utilized as electron acceptors in isolated complex-I-induced H_2_O_2_ formation; the production rate of H_2_O_2_ was partially prevented by rotenone, suggesting that water-soluble quinones react with oxygen when reduced at sites both downstream and upstream of the rotenone block. The one electron donor to oxygen in complex I is a non-physiological hydrophilic site [29,30] that reduces many quinones to their corresponding semiquinone forms, which are unstable and reduce oxygen to a superoxide. This mechanism is shared by several quinones, including anthracyclines [31], and the CoQ analog idebenone [32]. The hydrophilic, rotenone-insensitive site can apparently reduce oxygen to reactive oxygen species in the absence of intermediate acceptors [33]. Furthermore, many studies have reported that mitochondrial complex I is a major source of reactive oxygen species production in mitochondria [34,35] and localizes the oxygen-reducing site between the ferricyanide and the quinone reduction sites [36].

A recent study proposed two sites in a complex I reactive oxygen production model using the mitochondria of rat skeletal muscle. One site is in equilibrium with the NAD pool, seemingly the flavin of the FMN moiety, and the other site is dependent not only on the NAD redox state, but also on the proton motive force ∆p and the reduction state of the Q pool, seemingly a semiquinone in the Q-binding site [37]. Concurrently, Pryde and Hirst [5] devised a single mechanism of reactive oxygen species production by complex I under all conditions (during both NADH oxidation and reverse electron transfer) using bovine heart sub-mitochondrial particles. Generally, NADH-induced reactive oxygen species generation is prevented by complex I flavin-site inhibitors, but not by inhibitors of ubiquinone reduction and it is independent of the proton motive force (∆p). A reverse electron transfer by complex I in sub-mitochondrial particles, driven by the oxidation of succinate and the ∆p generated by ATP hydrolysis, reduces the flavin, leading to NAD^+^ and O_2_ reduction. However, similar to forward electron transport, reverse electron-transfer-stimulated reactive oxygen species generation is prevented by ubiquinone reduction and flavin site inhibitors. The potential dependence of NADH-stimulated reactive oxygen species generation (set by the NAD^+^ potential) matches with that of reverse electron-transfer-stimulated reactive oxygen species generation (set by the succinate potential and ∆p), and they both match the potential dependence of the flavin. Hence, the study suggested that NADH- and reverse electron-transfer-stimulated reactive oxygen species are generated by the flavin.

While mitochondrial complex I has been shown to be a key source of reactive oxygen species, and also a remarkable contributor to cellular oxidative stress in mitochondria, its ability to produce reactive oxygen species may vary under different oxidation–reduction pressure conditions [38]. In particular, a study on bovine heart mitochondria showed that the concentration and ratio of NAD^+^ and NADH regulate the production rate of reactive oxygen species [39]. For instance, at a lower NADH level, rotenone seemed to prevent the generation of reactive oxygen species in coupled sub-mitochondrial particles from the heart of beef. So, this phenomenon excludes a crucial function of complex I in reactive oxygen species under a lower reduction state of the iron–sulfur clusters of complex I. However, at a higher NADH level, rotenone induced reactive oxygen species generation. Recently, it was discovered that, with the exception of the concentration and ratio of NAD^+^ and NADH, many other factors also control the production of reactive oxygen species. For example, in reverse electron transport, the generation of reactive oxygen species is influenced by changes in the concentration of O_2_, the magnitude of the proton motive force, and the redox states of the co-enzyme Q (CoQ) and NADH pools [35]. Such a condition simulates the state of reduction of complex I when the respiratory chain is impaired.

Collectively, mitochondrial complex I generates large amounts of free radicals using two biological mechanisms: (1) a high ratio of NAD^+^/NADH leads to a reduction of the FMN site on complex I; and (2) electron transport to the CoQ pool coupled with a high protonmotive force ∆p leads to reverse electron transport. Although the site at which free radicals are generated during reverse electron transport has not been identified, the rate at which free radicals are generated under reverse electron transport appears to be the highest that can occur in mitochondria [37,39]. Furthermore, the mechanisms underlying the generation of free radicals that have been developed by the sub-mitochondrial particles, the isolated enzyme, and the coupled membrane vesicles need to be tested on mitochondrial complex I.

## 5. Complex II and the Generation of Free Radicals

The question of whether complex II, which is also known as succinate dehydrogenase, is a major source of reactive oxygen species remained controversial for many decades. Previous studies on isolated mitochondria and cells showed that complex II was not recognized as a remarkable contributor to reactive oxygen species until it experienced mutation [6,40]. Recently, the generation of reactive oxygen species from complex II of mitochondria has gained extensive scientific interest. Since then, many studies executed on sub-mitochondrial particles and intact isolated mitochondria have demonstrated that complex II, under specific conditions, and particularly when it is supplied with a high succinate concentration (almost 5 mM), produces a considerable amount of reactive oxygen species [41,42]. Interestingly, a recent study on skeletal muscle mitochondria of rats demonstrated that complex II produces reactive oxygen species in both the forward and reverse reactions as well as with a low and a high concentration of succinate [43,44]. Additionally, the authors suggested that complex II generates reactive oxygen species in the reverse reaction, for instance when electrons are provided from the reduced ubiquinone pool, and in the forward reaction, for instance when the electrons are supplied from succinate. Furthermore, they proposed a mechanism for the production of reactive oxygen species through complex II that entirely depends upon its possession of the carboxylate binding site and the enzyme reduction state; e.g., reactive oxygen species are produced when the binding site for carboxylate is not occupied and the flavin is reduced. Finally, it has been demonstrated that the complex-II-associated rates approach or exceed the maximum rates of complexes I and III, indicating that complex II may be an important contributor to the physiological and pathological generation of reactive oxygen species [24].

It has been shown that a functional loss of mitochondrial complex II can lead to succinate accumulation and enhanced reactive oxygen species production in cells [45]. This appears to particularly be the case for mutation in the subunits of complex II, and it is believed that it is associated with certain diseases. Mitochondrial complex II contains four subunits: SDHA, SDHB, SDHC, and SDHD. Mutation is mostly associated with SDHB, SDHC, and SDHC, while mutation in SDHA rarely occurs. A loss of SDHB, SDHC, and SDHD would allow for the acceptance of an electron, but not progression along the respiratory chain, and consequently may enhance the production of reactive oxygen species [46]. Ishii et al. [47] suggested that a mutation in the SDHC subunit of complex II in fibroblasts of a transgenic mouse and conditional transgenic mice stimulates dysfunction of the respiratory chain in mitochondria and enhances reactive oxygen species’ generation. Likewise, in a study on hamster fibroblasts, the authors observed that SDHD is also responsible for the production of reactive oxygen species [48]. Further, a mutation in SDHC of complex II triggered hypersensitivity that enhanced the concentration of oxygen in Caenorhabditis elegans, and stimulated the production of reactive oxygen species that was found in succinate sub-mitochondrial particles and fueled mitochondria from the mutant Caenorhabditis elegans [49,50].

To summarize, a growing amount of evidence suggests that mitochondrial complex II is an important modulator of reactive oxygen species generation under different physiological conditions. Hence, it can adapt different functions as a generator or regulator of reactive oxygen species depending on the supply of substrate, etc. [45,46]. Complex II’s biological functions need a rigorous analysis to identify the source of reactive oxygen species. This will be useful to provide a clear understanding of complex II’s functions in various pathophysiological conditions. Furthermore, the basic mechanisms by which mitochondrial complex II generates reactive oxygen species should be further studied in various model organisms.

## 6. Complex III and the Generation of Free Radicals

Complex III is an important multi-subunit, membrane-bound enzyme that is essential to respiratory energy transduction pathways in various organisms. Apart from energy production, it is known to contribute a considerable number of reactive oxygen species [51,52]. A remarkable advancement has been made in our understanding of complex III’s biological role in the production of reactive oxygen species. Various studies have described the mechanism by which complex III generates reactive oxygen species. According to these studies, it can produce reactive oxygen species by both a forward and a reverse electron transfer.

The first report regarding the involvement of complex III in reactive oxygen species generation surfaced in 1970s. The study explored the role of complex III in reactive oxygen species production using mitochondria from a rat’s heart. It showed that antimycin A, an oxidative phosphorylation inhibitor, cannot entirely prevent electron transport from ubiquinol to cytochrome c: the antimycin-A-insensitive reduction of cytochrome c is mediated by reactive oxygen species [53]. Another study proposed a mechanism for electron transport and reactive oxygen species generation from complex III, and suggested that two electrons from ubiquinol oxidation would be transported to the cytochrome c, but by using two pathways. One of the electrons would be transferred to cytochrome c through the chain reactions such as from quinol oxidation center to the iron–sulfur protein and finally to cytochrome c1, while the second one would be delivered to O_2_ for the production of reactive oxygen species. Additionally, preventing the quinone reduction at the quinone reduction (Qi) center using inhibitor molecules (e.g., antimycin A) remarkably enhanced quinol oxidation (Qo) center mediated reactive oxygen species generation [54]. Further, loss of iron–sulfur protein abolishes reactive oxygen species generation, whereas the loss of cytochrome b retains production of reactive oxygen species [55,56]. Many studies investigated the impact of particular Qi and Qo center inhibitors e.g., antimycin A, myxothiazol, on reactive oxygen species generation described complementary reactions in detail [57]. For example, declining electron transfer rate between the hemes b H and b L of cytochrome b, or eradicating the following oxidation of both the hemes by the Qi center inhibitor antimycin A, caused accumulation of electrons on cytochrome b [58]. This resulted in the accumulation of semiquinone radical at the Qo center and to the escape of electrons to O_2_ to produce reactive oxygen species [57]. If a semiquinone radical is formed at the Qo center during the normal turnover of a complex III, reactive oxygen species generation is likely to be relatively low to reduce electron leakage and energy wastage. However, reactive oxygen species generation at the Qo center possibly become remarkable under specific conditions, such as a highly reduced quinone pool and presence of inhibitor molecules (e.g., antimycin A). These abnormal conditions may occur only in extreme physiological conditions and in situation of complex III damage [59,60]. A recent study proposed a mechanism for generation of reactive oxygen species, when a complex III undergoes damage during disease resulted in the opening of permeability transition pores, consequently malate/succinate-fueled mediated generation of reactive oxygen species from complex III due to activation of malic enzyme through increases in matrix [Mg^2+^], [ADP] and [NAD^+^]. Further generation of reactive oxygen species in these physiological conditions is related to Mg^2+^ dependent NADH production by malic enzyme. For maximal production of reactive oxygen species, the production rate of NADH has to be almost equal or below that of NADH oxidation, as further increase in NADH elevate ubiquinol-related complex III reduction beyond the optimal range for reactive oxygen species generation [61].

The production of reactive oxygen species by reverse electron transfer pathway has gained attention in the current decade. Consequently, many studies were conducted to explore the mechanism and rate of reactive oxygen species generation by this pathway. Most of these studies suggested that partial oxidation of the quinone pool in a physiologically relevant condition remarkably enhances the rate of reactive oxygen species generation by antimycin A inhibited complex III [62,63]. Another study on sub-mitochondrial particles showed that complex III mediated reactive oxygen species generation is higher, when complex II activity is inhibited by its inhibitors (i.e., oxaloacetate or malonate), linking quinone pool redox state to reactive oxygen species generation by the Qo center of complex III. They inferred that produced reactive oxygen species was generated at the Qo center via reverse electron transfer from reduced heme b L of cytochrome b to O_2_ by semiquinol [64]. Afterwards, Quinlan et al. [42] argued that this effect is perhaps due to the redox state of hemes b H and b L of cytochrome b, which are highly sensitive to the membrane potential and redox state of quinone pool. Similarly, studies using bacterial model provided evidences that reactive oxygen species generation at the Qo center also used reverse electron transfer from reduced heme b L of cytochrome b, later on Sarewicz et al. [7] proposed a mechanism that shows transport of the Fe/S protein cluster from the Qo center enhanced reactive oxygen species production, while its stagnation reduced the production at Qo center.

Taken together, the exact mechanism for production of reactive oxygen species at the Qo center has still remained to elucidate and is currently a matter of debate. Furthermore, remarkable amount of reactive oxygen species from the Qo center have been measured under non-native conditions, for example, in the presence of antimycin A (inhibitor of the Qi center) [53,57]. Therefore, the basic question that remains to be explored is which physiological processes or factors promote production of reactive oxygen species from the Qo center in vivo.

## 7. Other Cellular Components and Free Radicals

There is increasing evidence that besides mitochondria, cellular components and other factors are also involved in the production of reactive oxygen species. Peroxisome, endoplasmic reticulum phagocytic cells, neuro-inflammation, dopamine, genetic mutation, anti-oxidant depletion etc. may induce the reactive oxygen species in cells. Thus, contributing to degeneration of neurons by lipid peroxidation, DNA and protein oxidation [65,66].

## 8. Environmental Sources of Oxidative Stress and Neurodegenerative Disorder

Besides the endogenous factors, exogenous sources of reactive oxygen species also contribute in the pathogenesis of neurodegenerative diseases including Parkinson’s and Huntington’s disease etc. Multiple lines of evidence suggest the etiology of neurodegenerative disorders is multifactorial and comprised of an interaction between environmental factors and genetic predisposition. Migliorea and his coworkers [67] reviewed that the environmental exposure to air pollution, metals, herbicides, insecticides and diet are the major risk factors in pathogenesis of neurodegenerative diseases by stimulating oxidative stress. Hence, to understand the pathogenesis of neurodegenerative disorders exogenous factors are also equally important, however, they are beyond the scope of the present review. 

## 9. Physiological Functions of Mitochondria in Neurodegenerative Disease

**Figure 2:**
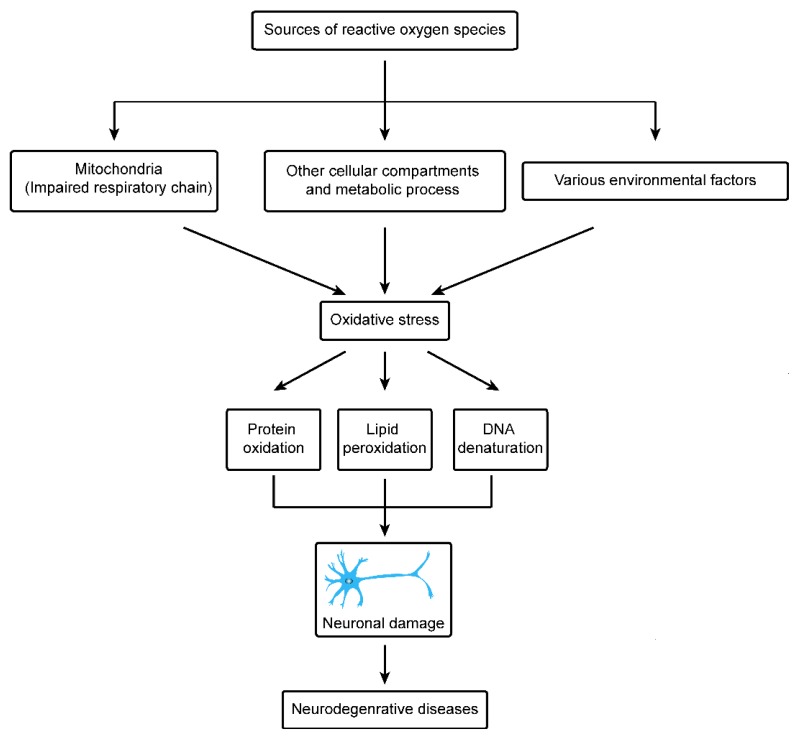
The causes of oxidative stress in neurodegenerative diseases.

In recent years, important advancements have been made in the field of medical science to understand initiating factors and cure of neurodegenerative diseases, however still it is a major cause of concern in the health profession. Neurodegenerative diseases can be divided into Parkinson’s disease, Alzheimer’s disease, multiple sclerosis, injury to the central nervous system by chronic low-grade hypoxia, motor neuron diseases, Wilson’s disease, Huntington’s disease and Freidreich’s ataxia [9]. Exact knowledge on the pathophysiological mechanisms is still insufficient and unclear in all of these neurodegenerative diseases. However, functional disorders of mitochondria have been reported to be a major cause in pathogenic process. A key challenge of modern neuroscience is to investigate and understand the level to which these variations in the functions of mitochondria depict the level (primary or secondary components) of the pathophysiological process, further to understand the fundamental biological pathways which lead to the initiation and development of neurodegenerative disease. A recent study suggested that mitochondria play critical functions in most neurodegenerative disorders. The mitochondria are mainly involved in ATP generation, reactive oxygen species generation, antioxidant activity and Ca^2+^ buffering. Neurons being high energy demanding cells are closely related to maintenance, dynamic activity, and functions of mitochondria. In most neurodegenerative diseases, mitochondrial dynamics and activities are impaired, causing low ATP production, high levels of reactive oxygen species, and apoptosis [68]. In this article our main focus is to review recent data regarding Huntington and Parkinson’s disease and the role of mitochondria in progression of these neurodegenerative diseases, particularly through generation of reactive oxygen species (Figure 2).

## 10. Mitochondria and Huntington’s Disease

Huntington’s disease is a detrimental neurodegenerative disorder, and people from the United States of America and Australia are highly affected by this disease, while a lower number of cases are also reported from China, Africa, Japan and other countries [69,70]. This disease is characterized by the irregular expansion of CAG trinucleotide repeats in exon 1 of the gene, which resides on the chromosome 4 and translates into an abnormal huntingtin protein. Healthy individuals usually contain 6 to 35 repeats of CAG, while this number is observed more than 36 repeats in patients [71]. Medically, Huntington’s disease is caused by dysfunction of motor neurons, psychiatric disturbances, and cognitive decay, consequently causing the patient to experience progressive muscle loss and brain dysfunction [72,73]. This disorder is commonly fatal within 15 to 20 years after disease onset.

Emerging evidence suggests that mitochondrial dysfunction is associated with the pathogenesis of Huntington’s disease. Panov et al. [74] observed mitochondria extracted from lymphoblasts of Huntington’s disease patients contain lower mitochondrial membrane potential compared with the control. Likewise, they found similar characteristics in mitochondria isolated from the brains of transgenic mice that transcribe mutant huntingtin protein. This physiological defect led the initiation of behavioral and pathological abnormalities. Based on these observations, they concluded that calcium abnormalities of the mitochondria arises at the onset of Huntington’s disease pathogenesis and probably is the direct influence of the huntingtin protein mutation on the tissues and organelle; however, it remains undiscovered exactly how the huntingtin mutant protein elicits its harmful effects. Mattson et al. [75] reviewed that mitochondrial-mediated oxidative stress, perturbed homeostasis of Ca^2+^, and apoptosis contribute to the onset of Huntington’s disease.

A recent study further highlighted this mechanism and demonstrated that the functional impairment of mitochondria and dysregulation of transcription are involved in Huntington’s disease pathogenesis. For example, Cui et al. [76] suggested that the mutant huntingtin protein causes perturbation of mitochondrial functions by preventing expression of PGC-1α, which controls different metabolic processes, including respiration and mitochondrial biogenesis. Crossbreeding of PGC-1α knockout mice with Huntington’s disease knock in mice lead to enhanced neurodegeneration of striatal neurons and motor abnormalities in mice with Huntington’s disease. Whereas, expression of PGC-1α partially reverses the toxic effects of mutant huntingtin in cultured striatal neurons. 

## 11. Mitochondria and Parkinson’s Disease

Parkinson’s disease is one of the most predominant neurogenerative diseases. Clinically, it is characterized by apoptotic loss of dopaminergic neurons, resulting in subsequent and gradual loss of muscle control. This disease is diagnosed by resting tremors, rigidity of muscle, change in gait and speech, postural instability, depression, fatigue, anxiety, sleep disturbances, and decline in dementia and cognition. Premature death of patients occurs due to complications e.g., pneumonia, injuries etc. [77]. Parkinson’s disease is prevalent in the adult population, usually in people over 65 years. The male population is approximately 1.5–2 times more susceptible to this disease than the female population [78].

There are multiple lines of evidence that link functional disorders of mitochondrial complex I and dopaminergic neurons in Parkinson’s disease [79]. A most appealing model of this disease was discovered in the 20th century, which described how MPTP (1-methyl-phenyl 4-phenyl-1,2,3,6-tetrahydropyridine) of the mitochondria is responsible for Parkinson’s disease [80,81]. Later on, several researchers independently discovered that sporadic Parkinson’s disease patients have reduced complex I in different brain areas, peripheral cells and neural and extra neural tissues and in cells (cytoplasmic hybrid), which are derived from Parkinson’s disease patients [70,82,83]. Another study on hybrid cell lines showed that complex I deficiency is correlated with the increased reactive oxygen species generation [84]. Further, many studies based on model organs and human neuroblastoma cells showed that in some patients, the disorder may reflect exposure to the plant extracted insecticide rotenone (complex I inhibitor) [85,86]. Exposure to low doses of rotenone induce apoptosis in human neuroblastoma cells. While, rotenone inhibition stimulates aggregation and accumulation of ubiquitin, α-synuclein, gradual oxidative damage and caspase-dependent cell death, which is the central mechanism in Parkinson’s disease pathogenesis. The binding site for rotenone appears irrefutably defined as mitochondrial complex I. It seems injury of cells is not a simple role of metabolic machinery inefficiency, as similar levels of ATP suppression stimulated by other poisonous chemicals such as glycolysis inhibition by 2-deoxyglucose completely failed to cause approximately the same cell injury. Rather, cells protect themselves using their antioxidant protection system, suggesting that oxidative stress is the primary mechanism for cell injury. Mitochondrial complex I is particularly susceptible to modification caused by oxidative stress, and thereby is a potential producer of reactive oxygen species [85].

A recent study on cell lines, human brain tissues and mice suggest that mutations in α-synuclein is associated with the pathogenesis and progression of Parkinson’s disease. Further, point mutations in α-synuclein are responsible for its decreased association with mitochondria associated membranes, coincident with a lower degree of apposition of endoplasmic reticulum with mitochondria, a reduce in mitochondria associated membranes function and increase in fragmentation of mitochondria [86]. Mutations in Parkin and PINK1 also contribute in the progression of different types of Parkinson’s diseases. The Parkin gene is highly expressed in brain tissues including the substantia nigra, and contains 12 exons, of which five (exons 3–7) are generally deleted and cause the pathogenesis and progression of disease [87]. Likewise, PINK1 (PTEN-induced kinase 1) is a mitochondrially-located molecule and has a protective impact on a cell. A mutation in its kinase domain can make cells susceptible to oxidative stress, and is thereby involved in the progression of disease [88].

Besides the above-mentioned factors, many others have been reported to be involved in dysfunction of mitochondria associated membranes. However, these factors were identified in other brain associated disorders, which is beyond the scope of our review. In short, c-secretase activity itself is highly enriched in a sub-compartment of the endoplasmic reticulum that is biochemically and physically connected to mitochondria. Mutation in the catalytic components of c-secretase (Presenilin-1 and -2) increase mitochondria associated membranes functions and communication between endoplasmic reticulum and mitochondria, which is the prominent characteristic of the familial and sporadic forms of Alzheimer disease. These results will help to understand calcium deregulation, mitochondrial dysfunction and oxidative stress in this disease and will also help to explore the contribution of this mutant in other brain associated diseases [89]. Similarly, Sigma 1 receptor plays a crucial biological role in the protection of motor neurons and its mutation lead to degeneration of motor neurons and caused Amyotrophic lateral sclerosis. A recent study reported that mutation occurs in highly conserved amino acid reside in the sigma receptor of *SIGMAR1* gene. The neuronal cells which express this mutant protein are less resistant to apoptosis stimulated by cellular stress [90]. Here, we garnered different contributing factors which are responsible for neurodegenerative diseases e.g., Alzheimer disease and that should be the focus of future studies to determine the biological role of these factors in brain associated diseases.

On the whole, cellular viability depends on the biological functions of mitochondria, and alterations in its biological functions can lead to cell functional abnormality and even cell death. Neuronal cells are especially susceptible to mitochondrial dysfunction due to their dependence for energy requirement on the mitochondrial metabolism. Dysfunction of mitochondrial respiration has been demonstrated to be involved in the pathogenesis of neurodegenerative diseases. However, currently the biological functions of mitochondria in neurodegenerative diseases appear to extend well beyond abnormalities in respiration. Although it remains to be explored whether alterations of mitochondria in neurodegenerative diseases constitute a primary or a secondary event, or are just part of a larger multifactorial pathogenic process.

## 12. Oxidative Stress and Parkinson’s Disease

The brain is the central organ in living organisms and is a major consumer of oxygen to provide uninterrupted supply of energy to approximately 86 billion neurons for their daily activities, it has been estimated it uses almost 20% of the basal oxygen (O_2_) from the total oxygen supplied to the human body [91,92]. This uptake of oxygen is utilized by mitochondria in respiratory chain to generate energy/ATP and reactive oxygen species are produced as by-product other than energy production, which is a major cause of DNA damage and one of the eminent characteristics of Parkinson’s disease [93,94]. 

Growing evidence suggests that oxidative metabolism has extensive functions in the Parkinson’s disease, as defective respiratory chain and mutation of mitochondrial DNA in the dopaminergic neurons are the most common features of Parkinson’s disease patients [95,96]. In the neurons of patients, metabolism of dopamine is greatly linked to oxidative stress and its biochemical degradation produces reactive oxygen species including hydrogen peroxide (H_2_O_2_) and other metabolic products. As the H_2_O_2_ is highly membrane permeable, these products enter into adjacent neurons where it has ability to interact with Fe^2+^ to hydroxyl radicals [97,98]. The remarkable elevation of iron in the redox active form in the dopaminergic neurons plays a key function in pathogenesis of Parkinson’s disease [99]. A recent study proposed a mechanism for iron accumulation in the brain neurons; functional disorder of mitochondria not only enhance reactive oxygen species generation but also decrease synthesis of iron–sulfur cluster and unorthodox activation of Iron Regulatory protein 1, a key regulator of iron homeostasis in a cell. This protein stimulates the accumulation of iron and hydroxyl radicals in a cell [100]. Iron ions can produce reactive oxygen species since ferrous ions (Fe^2+^) and ferric ions (Fe^3+^) can react with hydrogen peroxide and superoxide, respectively, in a chain reaction producing the hydroxyl free radical which together with oxidation of dopamine can induce neurotoxicity [101,102]. The iron accumulation in different areas of brain particularly in the dopaminergic neurons have been reported in the Parkinson’s disease, which suggest that iron mediated lipid peroxidation plays a major biological role in the development and progression of this disease [103,104]. For instance, the accumulation of byproducts of lipid peroxidation have been observed in the cerebral spinal fluid and serum of the patients [105]. A recent study suggested that the neurodegenerative process is increased, when neuro-melanin containing organelles accumulate high load of toxins and iron during aging. Additionally, the release of neuromelanin from degenerating neurons stimulates microglia and latter cause neurons death with further release of neuromelanin initiates a self-propelling mechanism of neuro-inflammation and neurodegeneration [106].

It has been shown with the onset of Parkinson’s disease metabolism of lipid peroxidation and proteins oxidation is increased in the dopaminergic neurons, and antioxidant level is also decreased. Additionally, thiobarbituric acid, malondialdehyde, and 4-hydroxy-2,3-nonenal level is also elevated in the substantia nigra of the patients [107,108]. Later, two-fold increase in protein oxidation in the substantia nigra was observed [109,110]. Furthermore, decrease in glutathione contents in human brain enhances the level of hydroxyl radicals in the patients [111]. Likewise, several experimental studies on human and model organisms suggest that glutathione peroxidase activities and glutathione contents are significantly decreased in the brain [112,113]. Multiple evidences, suggest that reduced activities of enzymatic and non-enzymatic antioxidants particularly in the dopaminergic neurons play crucial role in the progression and development of this disease [114,115,116]. Another study demonstrated that decrease in glutathione level and increase in oxidized glutathione contents is a common feature in the patients, whereas glutathione level is also decreased in the substantia nigra, due to loss of neurons [117].

## 13. Oxidative Stress and Huntington’s Disease

Besides major advancements in the field of molecular biology and medical sciences, the precise mechanism of neuronal death in Huntington’s disease remained mysterious. However, numerous reports suggest that oxidative stress play a major role in the development and progression of the disease [118,119]. In fact, susceptible brain neurons in the disease may not be able to cope the conditions of increasing reactive oxygen species. The higher level of reactive oxygen species may trigger intracellular cascades of oxidative stress through oxidizing DNA and protein, and inducing peroxidation of lipids in plasma membrane [120,121]. Mutant huntingtin proteins affect the mitochondrial activities by stimulating the opening of the mitochondrial permeability transition pore with the concomitant release of cytochrome c and the stimulation of the apoptotic mitochondrial pathway. This abnormal interaction also changes calcium buffering of mitochondria, further deteriorating mitochondrial disorder and enhancing reactive oxygen species production [122,123]. This mutant huntingtin protein interacts with Drp1, elevates GTPase Drp1 enzymatic activity, enhances abnormal dynamics of mitochondria and consequently, defective synaptic deficiencies and anterograde mitochondrial movement [124].

Although the oxidative damage has not been detected in the early stages of disease, yet it is assumed to have a crucial role in the progression of Huntington’s disease [125]. Many researchers observed a changed expression pattern and activity of nitric oxide synthase, antioxidants, and ascorbate in R6/2 HD and R6/1 transgenic mouse [126,127]. Reactive oxygen species generation is also enhanced in the striatum of transgenic mice [128], whereas, a recent study observed increase in lipid peroxidation in different mouse models [129]. Furthermore, decreased expression of antioxidants has also been detected in the model species, while increase of these antioxidant enzymes reduce the toxic effects of mutant huntingtin proteins in cultured neurons [130].

In the late stages of disease, level of oxidative stress plays a crucial role in the progression of Huntington’s disease. Mitochondrial dysfunction and impaired respiratory chain have been thought to involve in the reactive oxygen species mediated Huntington’s disease pathogenesis [131,132]. Brain tissues of the patients show a higher level of dysfunction in the components of oxidative phosphorylation [128]. Elevated oxidative stress markers and depletion of antioxidants in brain and peripheral tissues have also been observed in the patients [133]. Increase in reactive oxygen species in the patients causes the denaturation of other biomolecules, for instance, it has been proposed that reactive oxygen species mediated mitochondrial dysfunction, links with defective glucose metabolism in Huntington’s disease patients [134,135]. Another study suggested that the increase in reactive oxygen species level enhance lipid peroxidation and damage the cellular membranes, further, the higher lipid peroxidation is associated with lower glutathione content in Huntington’s diseases patients compared with the healthy subject [132,136]. A recent study on mice model organism proposed that oxidative stress greatly denature the DNA, and this denaturation accumulates at CAG repeats in a length dependent manner. Similarly, the rate of DNA damage is increased under the relevant physiological condition, for example reactive oxygen species mediated decrease in protein level and depletion of major base excision repair enzymes [137]. Later on, comparative study of striatal cells derived from Huntington’s diseases knock-in mice expressing mutant hunting versus wild cells indicated that higher level of reactive oxygen species greatly damages non-enzymatic antioxidants along with the other proteins [138]. Additionally, reactive oxygen species mediated abnormal metabolism of tryptophan further worsen the ongoing brain functional disorder [139,140,141].

Altogether, the mechanisms implicated in the pathogenesis and progression of Huntington’s and Parkinson’s disease have not been fully understood. However, there is increasing evidence that oxidative stress is one of the key events in the pathogenesis of these diseases. Apart from oxidative stress, failure of antioxidant enzymes in neurodegenerative disease patients plays a crucial role in neuro-degeneration. In the human brain, reactive oxygen species are mainly produced by mitochondrial dysfunction, dopamine metabolism and inflammation of neurons. Thus, the different protective mechanisms implicated in the modulation of these biological processes are an interesting area of research focus in current years. Moreover, the study of Huntington’s and Parkinson’s disease related proteins in combination with experimental research using model organism has yielded substantial insights into the molecular pathways of neuro-degeneration and highlighted previously unidentified mechanisms by which oxidative stress contributes to these diseases.

## 14. Conclusions

Neurodegenerative diseases are life-threatening disorders, rapidly spreading in the aged population, with the number of cases rapidly increasing worldwide. These diseases impose a substantial health burden both on the patients, their families and society. The mechanisms implicated in the pathogenesis and development of neurodegenerative diseases have not yet been completely understood. However, some biological mechanisms have been proposed for the development and pathogenesis of these diseases, of which functional disorders of mitochondria and oxidative stress are key mechanisms considered to be involved in the progression of these diseases. In brain tissues, reactive oxygen species are generated from mitochondrial disorders, dopamine metabolism and inflammatory neurons. Therefore, protective biological mechanisms, which contribute to the modulation of these biological processes, are an important area for future studies. Based on the results of previous studies, various therapeutic approaches such as restoring functions of mitochondria and reducing oxidative damage have been developed. However, despite encouraging outcomes in model organisms, many clinical trials have failed to describe an influence on the development of disease. Failures from these approaches analyzed so far should guide future, newer strategies.
